# Supplementary material for: Multiparametric platform for profiling lipid trafficking in human leukocytes
Source: Cell Rep Methods. 2022 Feb 8;2(2):100166. doi: 10.1016/j.crmeth.2022.100166 (PMC9017167; doi:10.1016/j.crmeth.2022.100166)
Supplement: Document S1. Figures S1–S6 — and Tables S1–S3 [file mmc1.pdf]

Cell Reports Methods, Volume *2*

Supplemental information

Multiparametric platform for profiling lipid trafficking in human leukocytes

Simon G. Pfisterer, Ivonne Brock, Kristiina Kanerva, Iryna Hlushchenko, Lassi Paavolainen, Pietari Ripatti, Mohammad Majharul Islam, Aija Kyttälä, Maria D. Di Taranto, Annalisa Scotto di Frega, Giuliana Fortunato, Johanna Kuusisto, Peter Horvath, Samuli Ripatti, Markku Laakso, and Elina Ikonen

Nucleotide change	Effect on protein	LDL-c mmol/l	Cholesterol-lowering medication	Age, years	BMI, kg/m^2
c.925_931del	p.(Pro309Lysfs*59)	2.01	Lipcut 20 mg, Ezetrol 10 mg	76	22.5
c.1876G>A	p.(Glu626Lys)	3.80		74	26.1
c.1784G>A	p.(Arg595Gln)	2.28		64	26.9
c.1876G>A	p.(Glu626Lys)	2.85	Lipitor 20 mg	63	23.8
c.1721G>T	p.(Arg574Leu)	3.47		61	23.8
c.925_931del	p.(Pro309Lysfs*59)	4.77		60	26.9
c.2375T>C	p.(Ile792Thr)	2.98		66	23.4
c.925_931del	p.(Pro309Lysfs*59)	5.59	Atorvastatin 80 mg Ezetrol 10 mg	73	26.2
c.925_931del	p.(Pro309Lysfs*59)	4.88	Simvastatin 20 mg	66	26.6
c.1085A>C	p.(Asp362Ala)	1.89	Crestor 10 mg	69	34.0
c.925_931del	p.(Pro309Lysfs*59)	3.72	Atorvastatin 40 mg	70	23.2
c.607C>T	p.(His203Tyr)	2.49	Lipcut 40 mg	77	33.8
c.1784G>A	p.(Arg595Gln)	1.83	THRIVE trial	73	28.6
c.1876G>A	p.(Glu626Lys)	2.19		61	22.7
c.1784G>A	p.(Arg595Gln)	3.41	Rosuvastatin 40 mg	58	28.6
c.796G>A	p.(Asp266Asn)	3.31	Atorvastatin 40 mg	59	24.7
c.1876G>A	p.(Glu626Lys)	1.73	Atorvastatin 40 mg	74	28.6
c.925_931del	p.(Pro309Lysfs*59)	3.84	Atorvastatin 80 mg	59	26.4
c.1876G>A	p.(Glu626Lys)	2.14	Lipcut 20 mg	67	25.6
c.925_931del	p.(Pro309Lysfs*59)	2.80	Rosuvastatin 40 mg	71	27.2
c.1335C>A	p.(Asp445Glu)	4.10		58	29.0
c.974G>A	p.Cys325Tyr	6.76		44	23.1
c1739C>T	p.Ser580Phe	5.21		29	19.0

Supplementary Table 1: Characteristics of heterozygous familial hypercholesterolemia (He-FH) patients included in this study, related to Figure 1G, H and Figure 2. This table lists *LDLR* nucleotide and amino acid changes for each patient, and provides additional information such as age, cholesterol-lowering medication, body mass index (BMI)(kg/m^2) and LDL-cholesterol (LDL-c).

	**Age** year	**BMI** kg/m2	**Hip-circ.** cm	**TC** mmol/l	**LDL-c** mmol/l	**HDL-c** mmol/l	**TG** mmol/l	**Apo-A1** mmol/l	**Apo-B** mmol/l
**nLDL-c** n = 19	58.8 ±10.0	25.3 ±4.3	99.0±10.3	4.5 ±0.7	2.3 ±	1.7 ±0.1	1.3 ±1.5	1.7 ±0.4	0.6 ±0.1
**hLDL-c** n = 20	61.3 ±13.4	27.1 ±2.6	101.3 ±7.7	8.1 ±1.2	5.8±1.1	1.6 ±0.4	1.6 ±0.5	1.6 ±0.3	1.5 ±0.3
p-value	n.s.	n.s.	n.s.	<0.001	<0.001	n.s.	n.s.	n.s.	<0.001

Supplementary Table 2: Characteristics of FINRISK subgroups with normal LDL-c (nLDL-c) and elevated LDL-c (hLDL-c), related to Figure 3, 5, and 6. Mean ± standard deviation, is depicted for: Hip-circ. = Hip circumference, TC = total cholesterol, LDL-c = LDL-cholesterol, HDL-c = HDL-cholesterol, TG = triglycerides, Apo-A1 = Apolipoprotein-A1, Apo-B = apolipoprotein-B. P-values were calculated with Welch's t-test.

	β	SE	p	Adjusted $r2$	AIC	Weight in combined PRS
**LDL-C**						
Talmud's PRS	0.24	0.0060	< 0.0001	0.061	65102	0.084
LDpred PRS	0.29	0.0059	< 0.0001	0.091	64683	0.244
Combined PRS	0.30	0.0059	< 0.0001	0.098	64126	

Supplementary Table 3. Performance of PRSs for LDL-c in the entire FINRISK cohort, related to Figure 6: Comparison of the performance of Talmud’s 12-SNP PRS1, the LDpred PRS, and the PRS combining Talmud’s and LDpred PRSs for LDL-C in the FINRISK cohort with lipid measurements. Performances were estimated using linear regression with residual lipid measurements after adjusting for age and sex as the response. Weight in combined PRS refers to the regression coefficients of standardised Talmud’s and LDpred LDL-C PRSs estimated in the independent GeneRISK cohort (n = 4697). PRS, polygenic risk score. SE, standard error. LDL-C, LDL-cholesterol. TG, triglycerides. AIC, Akaike information criterion.

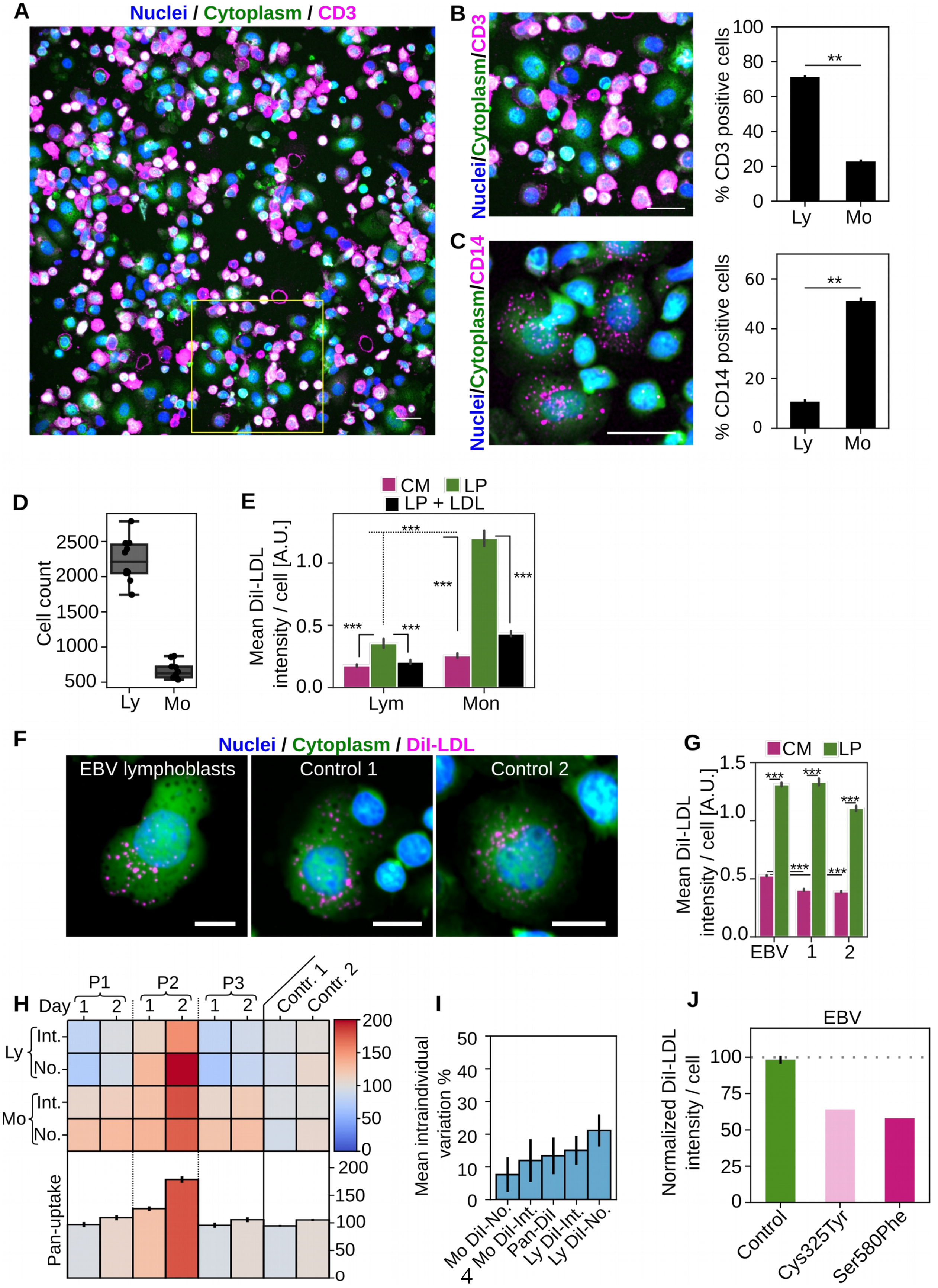
A
Nuclei / Cytoplasm / CD3
B
Nuclei/Cytoplasm/CD3
% CD3 positive cells
100
80
60
40
20
0
**
Ly
Mo
C
Nuclei/Cytoplasm/CD14
% CD14 positive cells
60
40
20
0
**
Ly
Mo
D
Cell count
2500
2000
1500
1000
500
Ly
Mo
E
CM
LP
LP + LDL
Mean Dil-LDL intensity / cell [A.U.]
1.0
0.5
0.0
***
Lym
Mon
F
Nuclei / Cytoplasm / Dil-LDL
EBV lymphoblasts
Control 1
Control 2
G
CM
LP
Mean Dil-LDL intensity / cell [A.U.]
1.5
1.0
0.5
0.0
***
EBV
1
2
H
Day
P1
P2
P3
1
2
Contr. 1
Contr. 2
Ly
Int.
No.
Mo
Int.
No.
200
150
100
50
0
Pan-uptake
I
Mean intraindividual variation %
50
40
30
20
10
0
Mo Dil-No.
Mo Dil-Int.
Pan-Dil
Ly Dil-Int.
Ly Dil-No.
4
J
EBV
Normalized Dil-LDL intensity / cell
100
75
50
25
0
Control
Cys325Tyr
Ser580Phe

Supplementary Figure 1) Detection of different white blood cell populations and performance studies for cellular LDL uptake, including specificity, comparison to EBV-lymphoblasts and intraindividual variation, related to Figure 1. **A**) Representative image of PBMC cells stained with DAPI (nuclei), CellMask Green (cytoplasm) and anti-CD3 antibodies (lymphocytes). **B**) Zoom in view of the yellow area in (**a**) and automated quantification of % CD3 positive cells for cells with a cytoplasm area below 115 μm^2 (designated as lymphocyte population) and above 115 μm^2 (designated as monocyte population), n = 2 controls, each containing PBMCs from 4 individuals; ±SEM. **c**) Representative zoom in image of anti-CD14 stained PBMCs and quantification of % CD14 positive cells in lymphocyte (Ly) and monocyte (Mo) populations as defined in (**B**), n = 2 controls, each containing PBMCs from 4 individuals; ±SEM. **D**) Box plot for lymphocyte and monocyte cell counts per well for a control containing PBMCs from 4 individuals; representative of eight independent experiments, n = 8 wells. **E**) Quantification of DiI-LDL intensities for lymphocyte and monocyte populations after treatment with control medium (CM, 10%FBS), lipid starvation (LP) or lipid starvation medium supplemented with 100 µg / ml native LDL during DiI-LDL uptake phase (LP+LDL). On average 5240 lymphocytes and 2580 monocytes were analyzed for each treatment per sample; ± 95%CI. **F)** DiI-LDL uptake in control EBV lymphoblasts (**EBV**) and PBMCs of two controls after lipid starvation. Representative images are shown. **G**) Quantification of mean DiI-LDL intensities in EBV lymphoblasts and monocytes from two controls after treatment with control medium (CM) or lipid starvation medium (LP), n > 20 000 for EBV lymphoblasts, and >16 000 monocytes from 10 independent experiments; ± 95% CI. **H**) Lymphocyte and monocyte cellular DiI-LDL intensities (Int), DiI-LDL organelle counts (No), and the pan-uptake score for three

individuals, sampled on two consecutive days; ±SEM, n = 8 wells (32 for pan-uptake) measured in two independent measurements. **I**) Average intraindividual variation for monocyte and lymphocyte uptake scores and pan-uptake, n = 3 individual persons; ±SEM. **J**) Quantification of cellular DiI-LDL intensities in EBV lymphoblasts from a control and two familial hypercholesterolemia patients (FH) after 72 h of lipid starvation; ±95% CI.

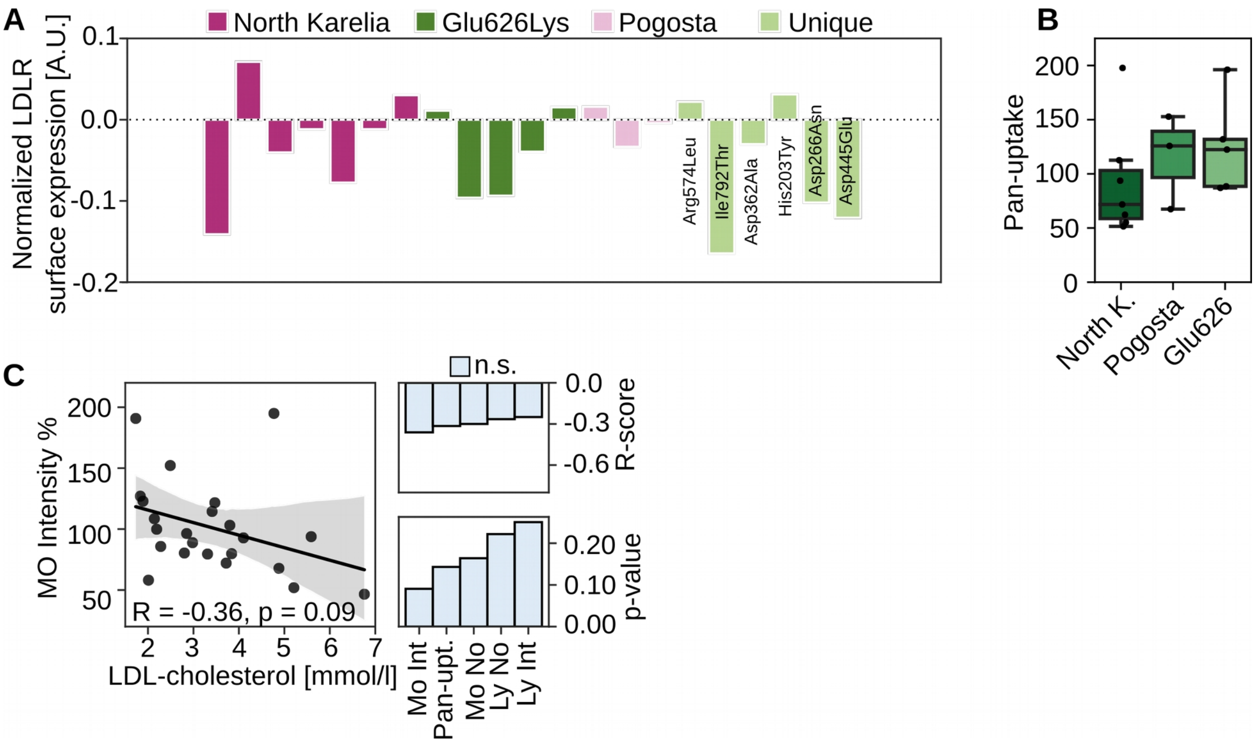

Supplementary Figure 2) LDLR surface expression for He-FH patients, summary of LDL uptake in FH subgroups and correlation of LDL uptake and circulating LDL-c for FH patients, related to Figure 2. A) Quantification of LDLR surface expression in monocytes after 24 h lipid starvation, relative to controls. On average, 1175 monocytes were quantified for each patient. **B**) Box plots for pan-uptake in heterozygous FH variant groups, North Karelia (North K.)(n = 7), Pogosta (n = 3) and Glu626 (n = 5). **C**) Correlation of monocyte DiI-LDL intensity and LDL-c concentration for heterozygous FH patients (n = 23) together with R- and p-values for all LDL uptake scores. Grey areas in scatter plots indicate 95% CI.

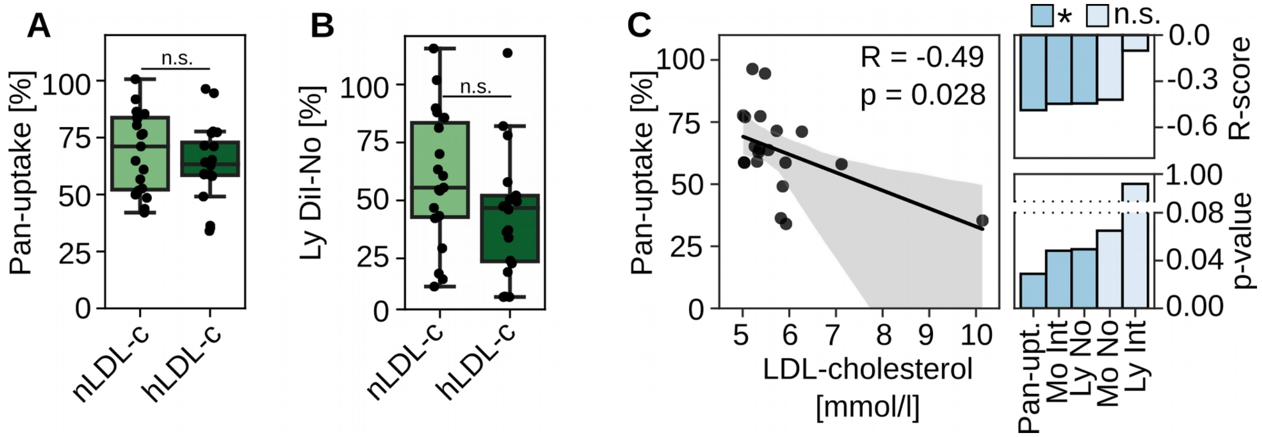

Supplementary Figure 3) Summary statistics for LDL uptake parameters of the FINRISK subjects and correlation of cellular LDL uptake and LDL-c for subjects with elevated LDL-c, related to Figure 3. Box plots for pan-uptake (**A**) and lymphocyte (Ly) DiI-LDL organelle numbers (DiI-No) (**B**) in individuals with normal (nLDL-c, LDL-c 2-2.5 mmol/l) and elevated LDL-cholesterol (hLDL-c, >5 mmol/l LDL-c); nLDL-c, n =19; hLDL-c, n =20, Welch's t-test. **C**) Correlation of pan-uptake with LDL-cholesterol for hLDL-c subjects, including R- and p-values for individual LDL uptake scores; n = 20. Grey areas in scatter plots indicate 95% CI.

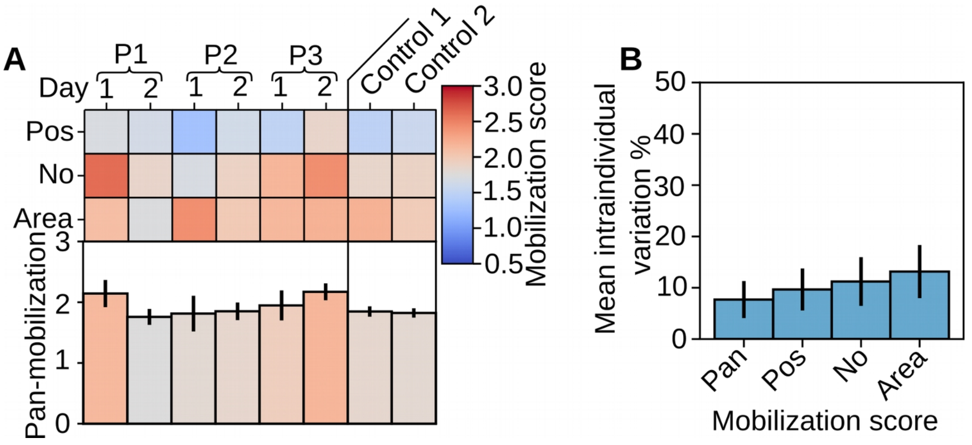

Supplementary Figure 4) Lipid mobilization assay performance, with quantification of intraindividual variation of lipid mobilization scores, related to Figure 4. A) Quantification of monocyte lipid mobilization scores LD-Pos, LD-No, LD-Area- and pan-mobilization as described in (**Figure 4G**) for three individuals sampled on two consecutive days; n = 4 wells (12 wells for pan-mobilization) from two independent measurements. **B**) Average intraindividual variation for lipid mobilization scores, n = 3 individual persons; ±SEM.

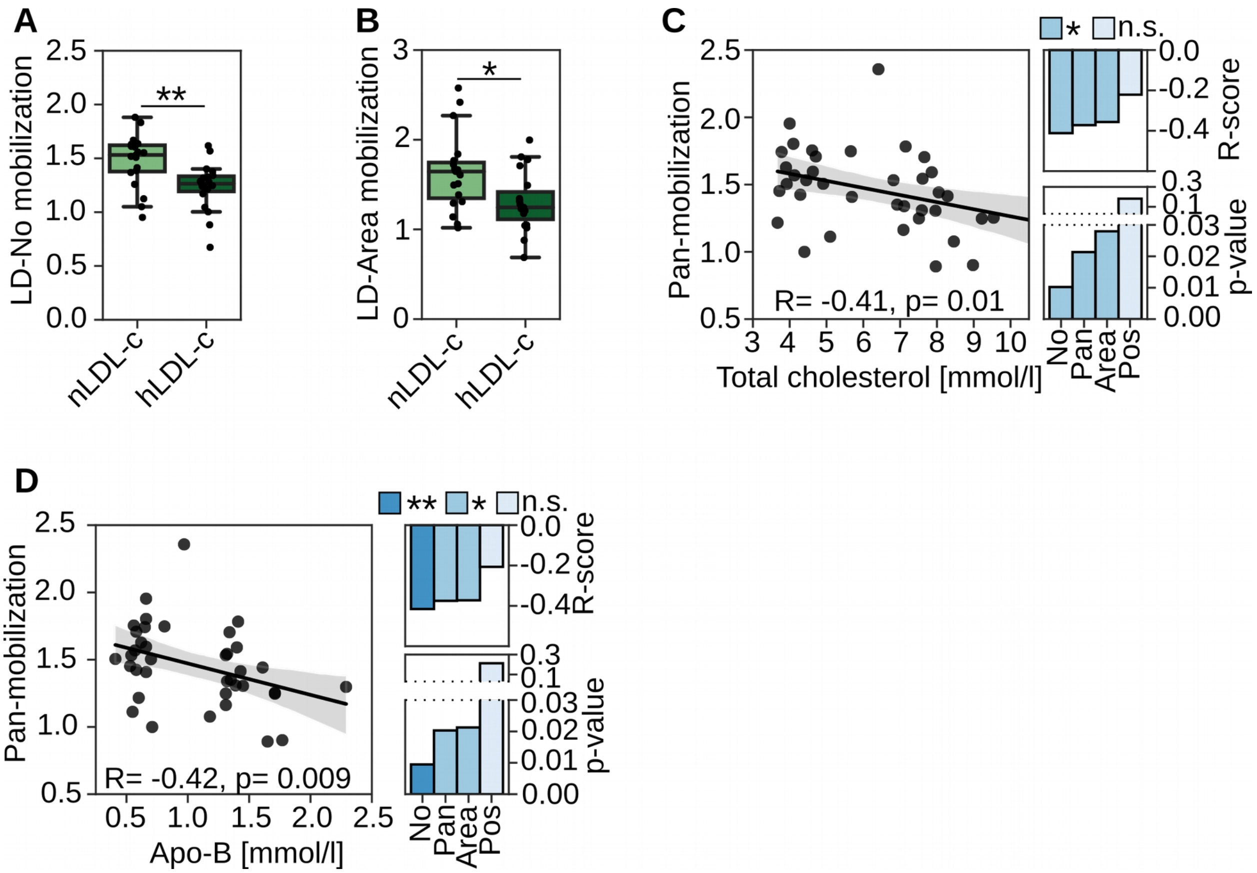

Supplementary Figure 5) Lipid mobilization summary statistics and correlation of lipid mobilization with total cholesterol and apolipoprotein-B (Apo-B), related to Figure 5. Box plots for lipid mobilization scores LD-No **(A)** and LD-Area **(B)** in individuals with normal (nLDL-c, LDL-c 2-2.5 mmol/l) and elevated LDL-cholesterol (hLDL-c, >5 mmol/l LDL-c); nLDL-c, n =19; hLDL-c, n =19; Student's t-test. ** p<0.01, **p<0.05. **C**) Correlation of pan-mobilization with total-cholesterol (mmol/l) and Apo-B (mmol/l) (**D**), including R- and p-values for all mobilization scores. Grey areas in scatter plots indicate 95% CI.

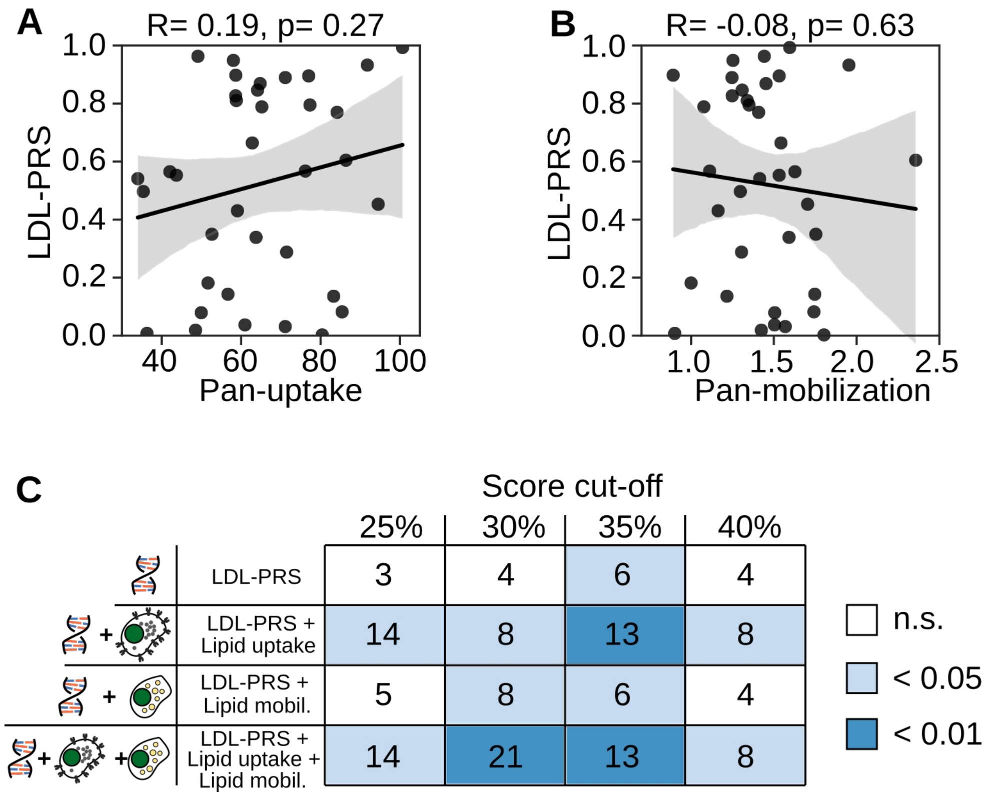

Supplementary Figure 6) Correlation of a polygenic risk score for high LDL-c (LDL-PRS) with cellular lipid uptake and mobilization, and calculation of odds ratios for different cut-off scores, related to Figure 6. Correlation of LDL-PRS with pan-uptake (**A**) and pan-mobilization (**B**) scores, n = 36. Grey areas in scatter plots indicate 95% CI. **C**) Odds ratio (OR) for 25, 30, 35 and 40% of the individuals with the highest LDL-PRS, double or triple hybrid scores and the remaining subjects, calculated with the Fisher's exact probability test. ORs significantly higher than 1 are indicated in light and dark blue, n = 36.
